# The Differential Effects of Acute Right- vs. Left-Sided Vestibular Deafferentation on Spatial Cognition in Unilateral Labyrinthectomized Mice

**DOI:** 10.3389/fneur.2021.789487

**Published:** 2021-12-08

**Authors:** Thanh Tin Nguyen, Gi-Sung Nam, Jin-Ju Kang, Gyu Cheol Han, Ji-Soo Kim, Marianne Dieterich, Sun-Young Oh

**Affiliations:** ^1^Department of Neurology, Jeonbuk National University Hospital & School of Medicine, Jeonju, South Korea; ^2^Department of Pharmacology, Hue University of Medicine and Pharmacy, Hue University, Hue, Vietnam; ^3^Department of Otorhinolaryngology-Head and Neck Surgery, Chosun University College of Medicine, Gwangju, South Korea; ^4^Research Institute of Clinical Medicine of Jeonbuk National University-Jeonbuk National University Hospital, Jeonju, South Korea; ^5^Department of Otolaryngology-Head and Neck Surgery, Gachon University of Medicine and Science, Graduate School of Medicine, Incheon, South Korea; ^6^Department of Neurology, Seoul National University Bundang Hospital & School of Medicine, Seoul, South Korea; ^7^Department of Neurology, University Hospital, Ludwig-Maximilians-Universität, Munich, Germany; ^8^German Center for Vertigo and Balance Disorders-IFB, University Hospital, Ludwig-Maximilians-Universität, Munich, Germany; ^9^Munich Cluster for Systems Neurology (SyNergy), Munich, Germany

**Keywords:** hemispheric dominance, labyrinthectomy, unilateral vestibular deafferentation, higher vestibular cognition, galvanic vestibular stimulation

## Abstract

This study aimed to investigate the disparity in locomotor and spatial memory deficits caused by left- or right-sided unilateral vestibular deafferentation (UVD) using a mouse model of unilateral labyrinthectomy (UL) and to examine the effects of galvanic vestibular stimulation (GVS) on the deficits over 14 days. Five experimental groups were established: the left-sided and right-sided UL (Lt.-UL and Rt.-UL) groups, left-sided and right-sided UL with bipolar GVS with the cathode on the lesion side (Lt.-GVS and Rt.-GVS) groups, and a control group with sham surgery. We assessed the locomotor and cognitive-behavioral functions using the open field (OF), Y maze, and Morris water maze (MWM) tests before (baseline) and 3, 7, and 14 days after surgical UL in each group. On postoperative day (POD) 3, locomotion and spatial working memory were more impaired in the Lt.-UL group compared with the Rt.-UL group (*p* < 0.01, Tamhane test). On POD 7, there was a substantial difference between the groups; the locomotion and spatial navigation of the Lt.-UL group recovered significantly more slowly compared with those of the Rt.-UL group. Although the differences in the short-term spatial cognition and motor coordination were resolved by POD 14, the long-term spatial navigation deficits assessed by the MWM were significantly worse in the Lt.-UL group compared with the Rt.-UL group. GVS intervention accelerated the vestibular compensation in both the Lt.-GVS and Rt.-GVS groups in terms of improvement of locomotion and spatial cognition. The current data imply that right- and left-sided UVD impair spatial cognition and locomotion differently and result in different compensatory patterns. Sequential bipolar GVS when the cathode (stimulating) was assigned to the lesion side accelerated recovery for UVD-induced spatial cognition, which may have implications for managing the patients with spatial cognitive impairment, especially that induced by unilateral peripheral vestibular damage on the dominant side.

## Introduction

Spatial cognition is the ability of an animal to keep track of its location in space by recalling where it has been, which is known as spatial memory (1, 2). Spatial cognition serves as the foundation for spatial navigation, which is the ability to move appropriately and purposefully through the environment (3, 4). Recent studies have demonstrated that the hippocampus plays a critical role in spatial memory consolidation and transitory storage (5, 6), and the peripheral vestibular organs are highly connected *via* several pathways including the thalamocortical and cerebellocortical pathways mediating head direction information (7–9). Therefore, the vestibular system is suggested to play a critical role in maintaining accurate spatial awareness (2, 4). Unlike other sensory cortices, the vestibular cortex consists of a network of several distinct and separate areas centered in the insular-opercular region (2, 4, 7). In addition, while other sensory systems are organized linearly, vestibular information from peripheral organs becomes multisensory, highly convergent, and highly multimodal when entering the central nervous system (10–13). Canal/otolith interactions in the brainstem and cerebellum at the first synapse, followed by visual-vestibular and proprioceptive-vestibular interactions throughout the central vestibular pathways, enable other sensory and motor signals to be integrated early with vestibular inflow (10, 14).

Numerous animal and human studies have demonstrated that bilateral vestibular loss (BVL) induces a prominent and long-lasting spatial memory deficit by disturbing vestibular-hippocampal interactions (3, 8, 15, 16). BVL results in bilateral hippocampus atrophy, which is associated with spatial memory deficits (17). Though to a lesser extent, unilateral vestibular deafferentation (UVD) has been shown to also impair spatial cognition (18–22). In addition, UVD in rodents has been revealed to lead to long-term changes in the neurochemical and electrophysiological properties of the hippocampus that contribute to spatial cognitive impairments (23–25). Galvanic vestibular stimulation (GVS) exhibits beneficial effects on spatial memory and navigation tasks by modulating the regularity of the firing rate of vestibular afferents (26–28). Further, improvement might in part be due to frequent activation of the vestibular hair cells and the neurons in the vestibular nuclei as well as the hippocampus (22, 29).

Although the peripheral and central vestibular systems are bilateral with ascending pathways on both sides, the multisensory cortical networks in each hemisphere are organized asymmetrically (30). In humans, vestibular information processing shows a hemispherical preference with a dominance determined by handedness (within the right hemisphere in right-handers and the left hemisphere in left-handers) (31–37). The right hemispheric preference for vestibular signal processing was observed in functional and structural imaging analyses showing stronger connectivity values, larger anatomical nodes, and higher functional vulnerability in the right hemisphere in right-handers (38). These findings are consistent with a well-documented superiority of the right hemisphere for visuospatial tasks and navigation (37, 39). In addition, the vestibular input to the ipsilateral hemisphere has a significant preponderance compared with the contralateral hemisphere (33, 37, 40). This lateralization of vestibular information processing was observed in ontogenetic older species. Vestibular processing in rodents was reported to be dominated by the left hemisphere regardless of handedness (41, 42). In rodents, the left-sided vestibular information is processed by more complex cortical and subcortical networks than the processing of information from the right-sided vestibular input (41). Considering that acute lesions in the dominant hemisphere can result in more severe symptoms, such as aphasia or neglect, than lesions in the non-dominant hemisphere do (43), it was speculated that the cognitive outcomes of left-sided or right-sided UVD can be different. However, only a few studies have investigated spatial cognition concerning the side of vestibular loss (44).

In the current study, we analyzed the differential effects of acute right- and left-sided UVD on the higher vestibular spatial cognition and locomotion reflecting hemispheric dominance using a mouse model with surgical labyrinthectomy. We also evaluated whether there was a differential effect of GVS on the deficits depending on the affected side.

## Materials and Methods

### Animals

**Figure 1:**
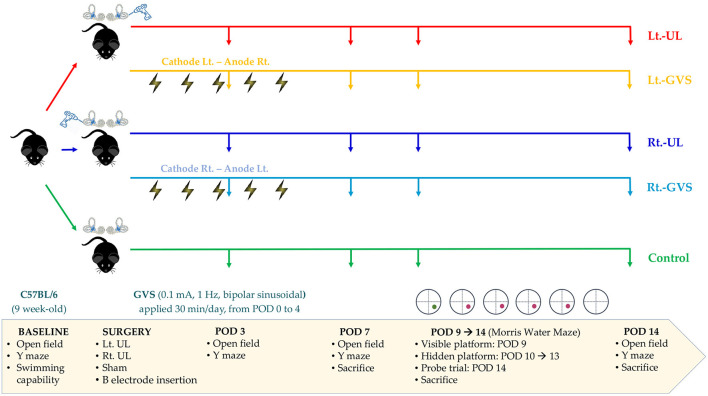
A schematic representation of the experimental design. Lt, left-sided; Rt, right-sided; UL, unilateral labyrinthectomy; GVS, galvanic vestibular stimulation; POD, postoperative day.

Sixty male C57BL/6 mice aged 9 weeks and weighing 20–25 g (Animal Technology, Koatech, Kyonggi-Do, Korea) were assigned randomly to five experimental groups: left-sided [Lt.- unilateral labyrinthectomy (UL) group, *n* = 12] and right-sided (Rt.-UL group, *n* = 12) UL groups, UL with bipolar GVS applications with cathode on the lesion side groups (Lt.-GVS group, *n* = 12 and Rt.-GVS group, *n* = 12), and the control group (*n* = 12) (Figure 1). Every effort was made to minimize the number and suffering of mice in the experiments. The mice were acclimatized to laboratory conditions for 1 week before the experiment started, housed separately, and kept in a controlled temperature and humidity room with free access to food and water.

Both the Lt.-UL and Rt.-UL groups underwent UL, which was carried out according to a surgery protocol as previously described (22, 45–49). The mice from the control group underwent sham surgery to expose the semicircular canal (SCC) without labyrinthectomy. We used surgical labyrinthectomy, which is relatively simple, reliable, and induces vestibular symptoms immediately after surgery; this approach also has a faster recovery than vestibular neurectomy and chemical labyrinthectomy (46, 50, 51). A 10-mm-long skin incision was made 5 mm behind the auricular sulcus to expose the bony labyrinth, and the muscle and soft tissues covering the temporal bone were dissected (45–48). After approaching the horizontal and posterior SCC, a small hole was made in the posterior SCC with a diamond otologic drill (0.5 mm in diameter; Strong 204, Saeshin Precision Co., Ltd, Shanghai, China) for perilymph leakage. Gentle suction was used to aspirate perilymph fluid for 3 min, and then the hole was filled with collagen (Helitene, Intergra Life Sciences Co., NJ, USA) to prevent further leakage. All the treated mice were anesthetized by continuous inhalation of isoflurane gas (Ifran, O_2_ 5 L/min, 2.0, Hana Pharm Co. Ltd., Kyonggi-Do, Korea) during surgery.

The animal procedures performed in this study were consistent with the Assessment and Accreditation of Laboratory Animal Care International guidelines and were approved by the Animal Care Committee of the Gachon University of Medicine and Science (IRB MRI2019-0008).

### Study Design

We evaluated the baseline levels of swimming capacity, open field (OF), and Y maze tests before labyrinthectomy. Mice that could swim were assigned randomly to the five treatment groups. OF and Y maze behavioral tests were used to measure locomotor activities and spatial cognition in each group on postoperative days (PODs) 3, 7, and 14 (Figure 1). The Morris water maze (MWM) training session was started on POD 9 and continued for five consecutive days, and the probe trial was performed on POD 14 (Figure 1). To minimize the time-of-day impact on the locomotor and exploratory behavior of mice (52), behavioral assessments were performed between 11:00 am and 3:00 pm.

To apply GVS, we implanted a button-type electrode near the bony labyrinths and connected it to a direct current (DC) shifted galvanic stimulator (A-M Systems Model 2200 Analog Stimulus Isolator) *via* a wire with an insulated section that passed through the skin, as previously described (22). A subthreshold, bipolar, sinusoidal GVS current of 0.1 mA and 1 Hz was generated by a computer-controlled stimulator and delivered over a 30-min session, once a day for 5 days. The Rt.- and Lt.-GVS groups were subjected to separate paradigms that ensured that the cathode was positioned on the lesion side, i.e., the cathode left-anode right (CLAR) configuration for the Lt.-GVS group and the cathode right-anode left (CRAL) configuration for the Rt.-GVS group. The mice in the control and UL groups were restrained by the same procedure as in the GVS groups but without current.

#### OF Task

**Figure 2:**
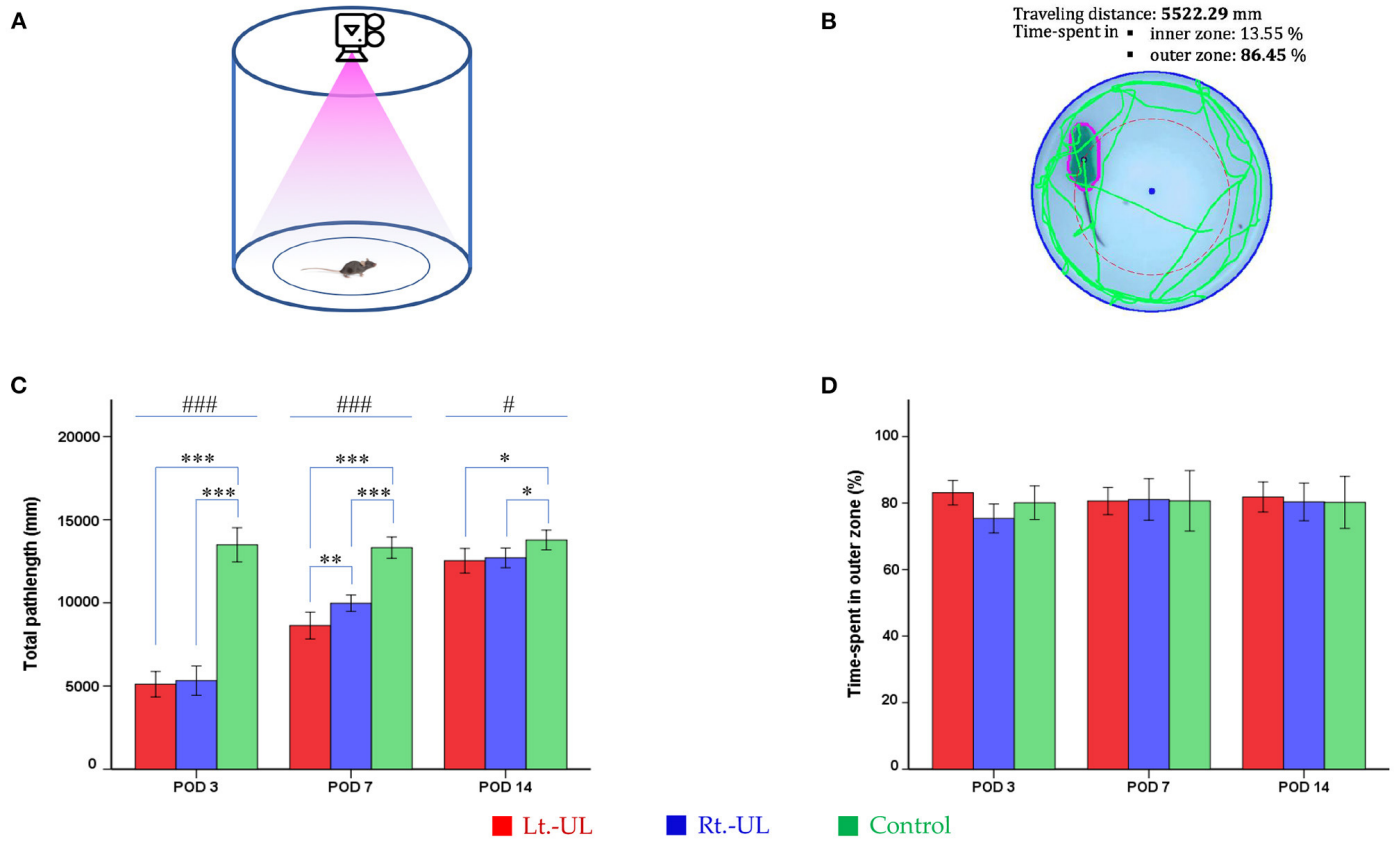
Evaluation of the locomotor activities of mice using an open-field task. The open field apparatus with an overhead camera and lighting support system **(A)**. The recorded images were processed with the digital video-based tracking system **(B)**. The total path lengths of the Lt.- unilateral labyrinthectomy (UL) and Rt.-UL groups were decreased in comparison to the control group on postoperative days (PODs) 3, 7, and 14. On POD 7, the total path length of the Lt.-UL group was significantly less than that of the Rt.-UL group **(C)**. The percentage of time spent in the outer zone, which is an indicator of anxiety, did not differ between the three groups **(D)**. Values are indicated as mean ± SD. Statistical significances were calculated using one-way ANOVA with *post-hoc* tests. *Significantly different between two groups; #significantly different between three groups; *, #*p* < 0.05; **p < 0.01; ***, ###*p* < 0.001.

Mice were tested for 2 min in an OF apparatus comprising a circular arena of a white plastic cylinder (37 cm diameter and 53 cm height) that was illuminated with red light from the top at the center of the apparatus (Figure 2A) (53, 54). The mice were introduced individually to the center and tracked by an overhead camera (HD 1080p C920, Logitech, Switzerland) at a sampling rate of 30 frames/s (Figure 2B). The locomotor activities of the mice were assessed as the total path length for the whole device ground (mm). The ground was divided into inner (central) and outer (peripheral) zones and the percentage of time spent in the outer zone was used as an indicator for anxiety (53, 54). The recorded images were processed with a customized analysis package (Figure 2B) (53, 54).

#### Y Maze

**Figure 3:**
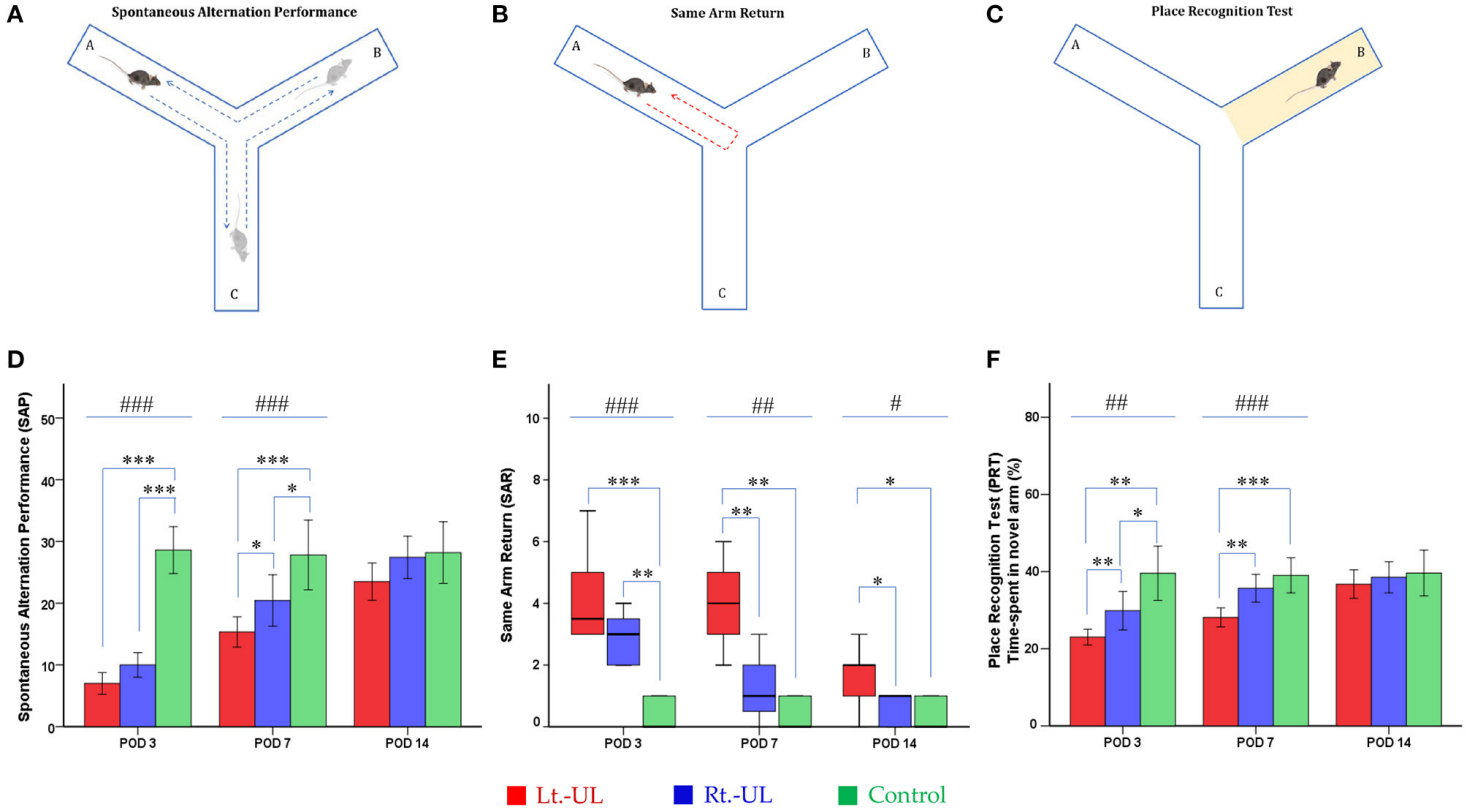
Evaluation of locomotor activities and spatial navigation in the Y maze test. The spontaneous alternation performance (SAP) **(A)** of Lt.- and Rt.-UL groups decreased on POD 3 and 7 compared with the control group. The SAP values of the Lt.-UL group were significantly lower than those of the Rt.-UL group on POD 7. **(D)** The same arm return (SAR) **(B)** was significantly increased in both Lt.-UL and Rt.-UL groups on POD 3 compared with the control group. While the SAR value of the Rt.-UL group decreased to the level of the control group on POD 7, the SAR of the Lt.-UL group increased on POD 7 and 14 compared with the control group. The Lt.-UL group showed significantly increased SAR compared with the Rt.-UL group on POD 7 and 14 **(E)**. The place recognition test (PRT) **(C)**, an indicator of spatial reference memory, was significantly decreased in both Lt.-UL and Rt.-UL groups compared with the control group on POD 3 **(F)**. While the PRT value of the Rt.-UL group improved to the level of the control group on POD 7, the value of the Lt.-UL group remained lower than that of the control group on POD 7. The Lt.-UL group showed significantly reduced PRT values compared with the Rt.-UL on PODs 3 and 7 **(F)**. The values of SAP and PRT were indicated as mean ± SD, and the *p*-values were calculated using one-way ANOVA with *post-hoc* tests. The values of SAR were indicated as median (quartile range), and the *p*-values were calculated using the Kruskal–Wallis test and the Mann–Whitney *U*-test. *Significantly different between two groups; #significantly different between three groups; *, #*p* < 0.05; **, ##*p* < 0.01; ***, ###*p* < 0.001.

A Y-shaped maze with three plastic arms (A, B, and C), 51 cm in length, 18 cm in width, and 32 cm in height with walls at an angle of 120° from each other, was used (Figures 3A–C). The maze was cleaned between the test runs to remove odors and traces that might have unexpected effects on the test outcome. Stress influences were eliminated by acclimatizing the mice for 1 h before the experiment to allow them to familiarize themselves with the room, smells, and noise (55, 56). Images of mouse activities throughout the task were captured by an overhead camera (30 frames/s) set at the center of the maze and used for behavioral analysis (56, 57). The mouse was introduced to the center of the maze and allowed to explore freely the three arms for 6 min. The following parameters were measured: *(i)* the spontaneous alternation performance (SAP), which is defined as entries into all three arms consecutively (e.g., ABC, BCA), to evaluate spatial working memory (55, 56) and *(ii)* the same arm return (SAR), which is defined as visiting the same arm repeatedly (e.g., if a mouse leaves arm A and then returns to arm A, one SAR is recorded); SAR reflects working memory error and typically correlates with disruption in spontaneous alternation (56–58). After several minutes of relaxation, spatial reference memory assessment was evaluated by blocking and unblocking the B arm (56). When the B arm was blocked, the mice could only move freely between the A and C arms for 3 min. After unblocking the B arm, the mice could move throughout the three arms for 6 min. The percentage of time spent in the B arm designated as the novel arm was used for the place recognition test (PRT), reflecting spatial working and reference memory (56, 57). The values of SAP, SAR, and PRT were measured in each group at four time points: baseline and PODs 3, 7, and 14 (Figure 1).

#### MWM

**Figure 4:**
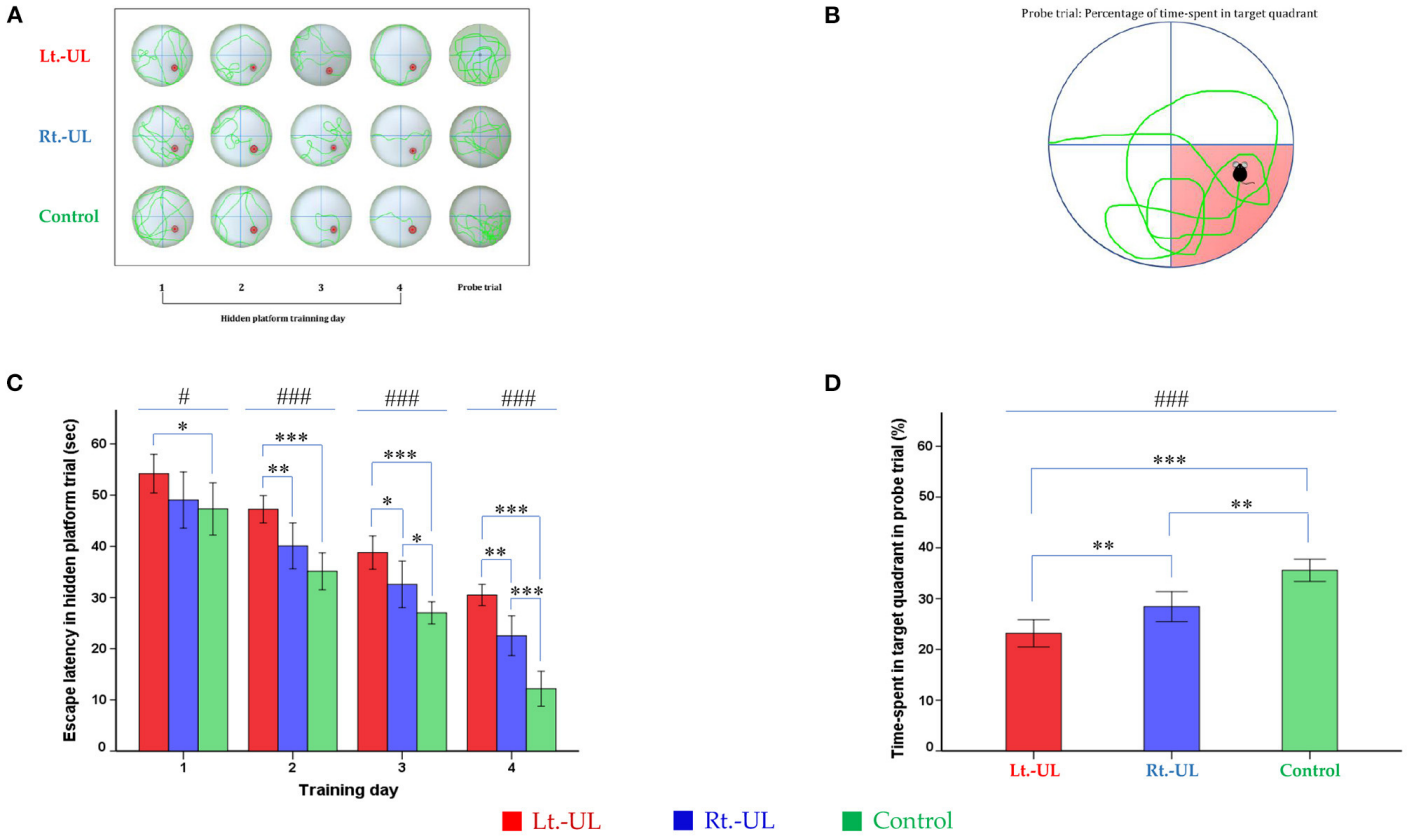
Evaluation of long-term spatial reference memory with the Morris water maze (MWM). The process of hidden platform training (POD 10–13) **(A)** and the probe trial on POD 14 **(B)**. Compared with the control group, the Lt.-UL mice showed longer escape latency from TD 1, 2, 3 to 4. The Rt.-UL mice showed reduced values compared with the control mice on the two last TDs. Subgroup analysis revealed that the Lt.-UL mice showed markedly longer escape latency than the Rt.-UL group in the last 3 days **(C)**. During the probe trial on POD 14, there was a decreased percentage of time spent in the target quadrant in both Lt.-UL and Rt.-UL groups compared with the control group. The Lt.-UL mice showed a lower percentage of time in the target quadrant values compared with the Rt.-UL mice **(D)**. The values are indicated as mean ± SD. Statistical significances were calculated using the one-way ANOVA with *post-hoc* tests. *Significantly different between two groups; #significantly different between three groups; *, #*p* < 0.05; **p < 0.01; ***, ###*p* < 0.001.

For the evaluation of spatial memory and navigation, we used the MWM, which uses a plastic circular water tank (175 cm diameter and 62 cm high; Jilong Frog Pool, Jilong International Co., Ltd, Hong Kong) with four starting locations of N, S, E, and W (Figure 4A) (59–61). A circular escape platform 15 cm in diameter was made of acrylic with a metal textured surface to provide traction on the top and placed in a fixed location at the center of the target quadrant (SE). The platform was attached to the manual laboratory scissor jack (4 × 4” Scientific Lab Laboratory Scissor Jack, Yosoo, Shenzhen Yibai Network Technology Co. Ltd, China) to make it easier to alternate between scenarios: visible platform, hidden platform, and no platform (1.5 cm above and 1.5 and 10 cm, respectively, below the surface of the water) (17). The ratio of the search area to target platform size related to task intricacy is appropriate for the 117:1 ratio of the MWM standard for mice (59). The water was made opaque by non-toxic odorless white paint, which helps to obscure the submerged platform and enables the software to locate the mice because of the contrast between the black body with the white background of the pool. A camera (HD 1080p C920; Logitech International SA, Lausanne, Switzerland) mounted in the center above the pool recorded the behavior of the mice throughout the experiment. The mice were acclimatized to the pool and escape platform before training on POD 8. The visible platform trial was conducted on POD 9, and the hidden platform trial was carried out on four consecutive days (POD 10–13). Each day, the mice were subjected to four trials after being lowered gently tail-first into the pool facing the wall at four starting points (N, S, E, W). The mice locate the escape platform based on visual cues [placement of a black triangle, red rectangle, green star, and blue circle on the surrounding walls (59, 60)] rather than on specific routes (internal self-motion cues) (59, 62). The mice were released at varying positions to exclude the turn-based trajectory to reach the platform, and they sought to use allocentric strategies to compute and remember an escape location defined by distal cues in the environment (6, 63). Each mouse was allowed 1 min to find and mount the platform. If a mouse failed to find the platform within the allotted time, it was guided to the goal and placed on the platform for 15 s. If the goal was reached, the mice remained in place for 10 s (59, 64). The mice were removed from the pool, dried, and placed in a warming cage for 5 min before returning to the home cage. The 20-min intervals between trials helped to eliminate the negative impact of fatigue on learning. The amount of time that elapsed before the animal climbed onto the platform to escape the water (escape latency) in a hidden platform training session, measured at a fixed starting location (position W) was recorded (Figure 4B).

The probe trial (no platform) was administered 24 h after the last training session. The mice were released at starting position W and swam freely for 1 min (60). The percentage of time spent in the target quadrant (SE quadrant) was measured and reflected spatial reference memory. A visible platform test was performed 30 min after the probe trial to assess the sensorimotor ability and motivation (60) that was indicated by the mean swim velocity = $\frac{path\;length\;\left(mm\right)}{escape\;latency\;\left(s\right)}$.

The deficits of spatial working memory were indicated by the reduction of SAP, which is driven by the innate curiosity of the rodents to explore novel environments and requires good spatial working memory to remember the arms that have already been visited to enter a less-visited arm (65), and increased SAR, which reflects working memory error and typically correlates with disruption in spontaneous alternation (56, 58). Spatial reference memory deficiencies were defined by a decrease in the percentage of time spent on the novel arm in the PRT, which required the capacity to recall the relationship between distal spatial signals to the arm needed to recognize the novel arm which had previously been blocked and not yet explored (56). The 24-h interval between the training session and the probe trial session of MWM was analyzed to assess long-term memory or consolidation process in the hippocampal-dependent spatial navigation and reference memory rather than the immediate and short-term effects of unilateral vestibular loss (17, 66–68).

### Statistical Analysis

All data were analyzed using SPSS Statistics version 23.0 (IBM Corp., Armonk, NY, USA). For each parameter, the normality of the distribution was assessed using the Kolmogorov–Smirnov test. The repeated measures ANOVA or Friedman Tests were used to analyze the interaction between surgical conditions—time as a first-level analysis. The parametric variables are shown as mean ± SD, and statistical significances were calculated using *post-hoc* one-way ANOVA accompanied by a test of homogeneity of variances (Levene test): *(i)* if *p* > 0.05, ANOVA test (between-group comparison) and least significant difference LSD test or Bonferroni test (multiple comparisons) were used; and *(ii)* if *p* < 0.0*5*, Robust test (between-group comparison) and Tamhane test (multiple comparisons) were used. The non-parametric variables are indicated as median [interquartile range], and the significant difference was determined using Kruskal–Wallis test (between-group comparison) accompanied by Mann–Whitney *U*-test or Wilcoxon signed-rank test (pairwise comparisons). The difference in the influence of GVS on the CLAR and CRAL models was analyzed by independent *t*-tests comparing the delta values of the Lt.-GVS and Lt.-UL to the respective delta values of the Rt.-GVS and Rt.-UL. All the tests were performed at a 0.05 level of significance.

## Results

During the acute phase after surgery, signs of UVD, such as spontaneous horizontal nystagmus beating toward the contralesional side, head-tilting, falling toward the ipsilesional side, disturbance in backward gait, and clockwise circling, were observed in both Lt.-UL and Rt.-UL groups. The control animals that underwent sham operations did not show these symptoms. It took ~2 days after UL for the mice to regain a stable posture and walk steadily. Based on this observation, we conducted all behavioral investigations starting from POD 3 when the mice were free from the limitations of motor coordination problems (Figure 1).

### Locomotion Following Acute Right- vs. Left-Sided UL in Mice

Both the Lt.-UL and Rt.-UL groups exhibited locomotor impairment during the OF test compared with the control group, as shown by the decreased total path length on POD 3 (*p* < *0.001*, ANOVA), POD 7 (*p* < *0.001*, ANOVA), and POD 14 (*p* < 0.0*5*, ANOVA). We compared the locomotion between the right- vs. left-sided UL groups and found no significant differences in the total path length (*p* = 0.655, LSD test, Figure 2C) and SAP (*p* = 0.103, LSD test, Figure 3D) during the hyperacute period of POD 3. However, on POD 7 during vestibular compensation, the Rt.-UL group showed a significantly increased total path length compared with the Lt.-UL group (9973.8 ± 533.02 mm vs. 8634.3 ± 767.52 mm, *p* < 0.0*1*, Bonferroni test, Figure 2C). Similarly, the SAP of the Rt.-UL group was significantly increased compared with that of the Lt.-UL group (*p* < 0.0*5*, LSD test, Figure 3D).

We further observed that the percentage of the time spent in the outer zone during OF did not differ between the UL and control groups, and the mice tended to remain near the wall as a normal phenomenon (Figure 2D). This behavior is interpreted as an indicator of anxiety (53, 69, 70), based on the assumption that the central area is more threatening for rodents than the periphery (44). This is supported by a decrease in time in the center following anxiolytic drug administration (71, 72). These findings suggest that the impact of anxiety on locomotor and spatial cognition in the current study was negligible.

### Spatial Cognition Deficits Following Acute Right- vs. Left-Sided UL in Mice

The alternation performance and spatial recognition/attention reflected by SAP and SAR during the Y maze were disrupted in both Lt.-UL and Rt.-UL mice during the acute phase. SAP, an indicator of spatial working memory as well as locomotor activity, was decreased on POD 3 in both Lt.- and Rt.-UL groups (both *p* < 0.001, Bonferroni test) compared with the control group (*p* < 0.001, ANOVA test). This reduction of SAP was observed until POD 7 in both Lt.-UL and Rt.-UL groups (Lt.-UL, *p* < 0.0*01*; Rt.-UL; *p* < 0.0*5*, Bonferroni test) compared with the control group (*p* < 0.0*01*, ANOVA test). It was recovered on POD 14 in both groups (Lt.-UL, *p* = *0.5*5; Rt.-UL, *p* = 0.715, LSD test). The subgroup analysis between the Lt.- and Rt.-UL groups showed no differences in alternating performance on POD 3. However, the Rt.-UL group alternated between the arms of the maze more frequently (an increased number of arm entries) compared with the Lt.-UL group on POD 7 (*p* = 0.034, LSD test) (Figure 3D).

The SAR was scored as cumulative returns into the same arm and suggests the degree of attentional difficulties during active working memory performance. The number of SAR was significantly increased in both the Lt.-UL and Rt.-UL groups on POD 3 (Lt.-UL, 3.5 [3–5.5] turns, *Z* = −2.796, *p* < 0.001; Rt.-UL, 3 [2–4] turns, *Z* = −2.898, *p* = 0.004, Mann–Whitney *U*-test) compared with the control group (0 [0–1] turns, χ_[2]_ = 11.985, *p* < 0.001, Kruskal–Wallis test). There was no significant difference between the Lt.-UL and Rt.-UL groups on POD 3 (*Z* = −1.636, *p* = 0.102, Mann–Whitney *U*-test) (Figure 3E). On POD 7, the Lt.-UL mice did not improve and showed persistently increased SAR (4 [2.75–5.25] turns, *Z* = −2.777, *p* = 0.005, Mann-Whitney *U*-test) compared with the control group (0 [0–1] turns). In contrast, the Rt.-UL group improved and exhibited decreased SAR (1 [0–2] turns, *Z* = −1.462, *p* = 0.144, Mann–Whitney *U*-test) to the level of the control group (χ_[2]_ = 11.362, *p* = 0.003, Kruskal–Wallis test) (Figure 3E). Increased SAR in the Lt.-UL mice persisted until POD 14 (2 [0.75–2.25] turns, *Z* = −2.001, *p* = 0.045, Mann–Whitney *U*-test), on which the Rt.-UL group showed decreased SAR (1 [0–1] turns, *p* = 0.575, Mann-Whitney *U*-test) compared with the control group. The between-group analysis revealed that the Lt.-UL mice showed significantly increased SAR compared with the Rt.-UL group on POD 7 (*Z* = −2.672, *p* = 0.008, Mann–Whitney *U*-test) and POD 14 (*Z* = −2.025, *p* = 0.043, Mann–Whitney *U*-test) (Figure 3E).

The PRT, which is an indicator of spatial reference memory, was significantly different between the groups during the acute periods of vestibular compensation. The mean time spent in the novel arm was significantly decreased in both Lt.-UL and Rt.-UL groups (Lt.-UL, 23.01 ± 1.95%, *p* = 0.005 vs. Rt.-UL, 29.87 ± 5.39%, *p* = 0.049, Tamhane test) compared with the control group (39.56 ± 5.66%, *p* = 0.001, Robust test) on POD 3 (Figure 3F). However, on POD 7 the Rt.-UL group had improved to the level of the control group (*p* = 0.111, LSD test). The value of the Lt.-UL group remained lower than that of the control group on POD 7 (Lt.-UL; 28.11 ± 2.33% vs. control; 39.03 ± 3.66%, *p* < 0.0*01*, Bonferroni test). The subgroup analysis revealed much lower values for the Lt.-UL mice for visiting the novel arm compared with the Rt.-UL mice on POD 3 (Lt.-UL; 23.01 ± 1.95% vs. Rt.-UL 29.87 ± 5.39%, *p* = 0.004, Tamhane test) and POD 7 (Lt.-UL; 28.11 ± 2.33% vs. Rt.-UL 35.66 ± 3.89%, *p* = 0.003, Bonferroni test). However, on POD 14 there were no differences in the values of PRT between the three groups (Figure 3F).

During the MWM, the escape latencies to find the hidden platform gradually decreased through the training sessions (Figures 4A–C). Longer values of escape latency to find the hidden platform indicate an inadequate acquisition of spatial memory and navigation. Differences between the groups were observed from training day (TD) 1 to 4 (Figure 4C). Compared with the control group, the Lt.-UL mice showed longer escape latency on every test day: TD 1 (*p* = 0.043, Bonferroni test), TD 2 (*p* < 0.001, Bonferroni test), TD 3 (*p* < 0.001, Bonferroni test), and TD 4 (*p* < 0.001, Bonferroni test). The Rt.-UL mice showed reduced values compared with the control mice on the two last TDs (32.56 ± 3.66 s vs. 27.0 ± 1.75 s, *p* = 0.013, Bonferroni test on TD 3; 22.53 ± 3.12 s vs. 12.17 ± 2.76 s, *p* = 0.001, Bonferroni test on TD 4) (Figure 4C). The subgroup analysis revealed that the Lt.-UL mice showed markedly longer escape latency than the Rt.-UL group on TD 2 (47.25 ± 2.54 s vs. 40.08 ± 3.62 s, *p* = 0.005, Bonferroni test), TD 3 (38.77 ± 3.09 s vs. 32.56 ± 3.66 s, *p* = 0.013, Bonferroni test), and TD 4 (30.49 ± 1.97 s vs. 22.53 ± 3.12 s, *p* = 0.001, Bonferroni test) (Figure 4C).

During the probe trial on POD 14, a decreased percentage of time was spent in the target quadrant in both the Lt.-UL (23.17 ± 2.56%, *p* < 0.001, Bonferroni test) and Rt.-UL groups (28.43 ± 2.38%, *p* = 0.001, Bonferroni test) compared with the control group (35.57 ± 1.76%, *p* < 0.001, ANOVA) (Figure 4D). The Lt.-UL mice exhibited a significantly reduced percentage of time spent in the target quadrant compared with the Rt.-UL mice (23.17 vs. 28.43%, *p* = 0.007, Bonferroni test) (Figure 4D). There were no significant differences in the mean swim velocity between the groups (*p* = 0.605, *ANOVA*), indicating that these MWM learning impairments were not specific to vestibulo-motor deficits (59, 73).

### Efficacy of GVS on Spatial Cognition Depending on the Lesion Side in UVD in Mice

Galvanic vestibular stimulation intervention paradigms were designed to stimulate the lesion side in each group by placing the CLAR for the Lt.-UL group and the CRAL for the Rt.-UL group. This bipolar GVS intervention demonstrated beneficial effects on the vestibular recovery such as vestibulo-ocular reflex (VOR), locomotion, and spatial cognition, as shown in our previous studies (22, 49).

**Figure 5:**
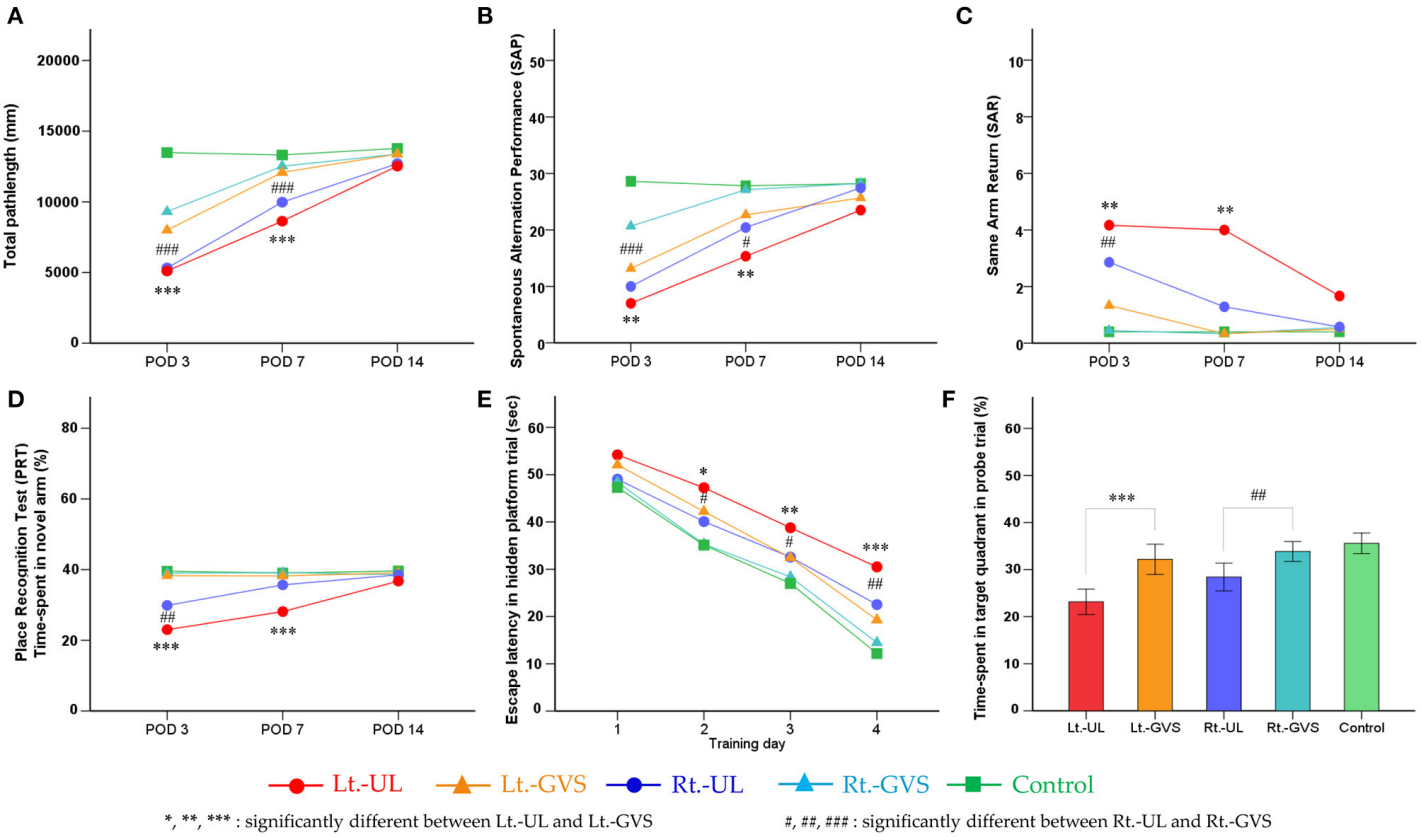
Effects of galvanic vestibular stimulation (GVS) on the recovery post-UL. Bilateral bipolar GVS was applied as paradigms of cathode left–anode right (CLAR) for Lt.-GVS and cathode right–anode left (CRAL) for Rt.-GVS. Locomotion was reflected by the total path length on PODs 3 and 7 **(A)** and by SAP on PODs 3 and 7 **(B)** were markedly improved after GVS applications in both groups. The subgroup analysis between the Lt.- and Rt.-GVS groups revealed that the GVS intervention was significantly effective in the Rt.-UL mice compared with the Lt.-UL mice on POD 3 on total path length **(A)** and SAP **(B)**. Short-term spatial memory and attention reflected by SAP, SAR, and PRT during the Y maze were significantly improved after bipolar GVS intervention in both groups. The GVS protocol exhibited positive effects on the recovery of SAP on PODs 3 and 7. SAR showed significant improvement after GVS intervention in both side groups on POD 3 and the left side group only on POD 7. The GVS intervention was significantly more effective in the Rt.-UL mice than the Lt.-UL mice on POD 7 **(C)**. PRT was significantly improved after GVS on POD 3 and POD 7. Subgroup analysis revealed that the GVS intervention was more effective in the Rt.-UL mice than the Lt.-UL mice on POD 3 and POD 7 **(D)**. Long-term consolidative spatial memory reflected by the escape latency in MWM was improved after GVS in both side groups on hidden platform training days (TDs) 2, 3, and 4. There was, however, no noticeable difference between the Lt.-GVS and Rt.-GVS groups **(E)**. The probe trial on POD 14 showed substantial improvement after GVS intervention in both side groups with a significant difference between the Lt.-GVS and Rt.-GVS groups **(F)**. Values are indicated as mean. Statistical significances were calculated using the one-way ANOVA with *post-hoc* tests, except the Kruskal–Wallis test combined with Mann–Whitney *U*-test for SAR. The difference in the influence of GVS on CLAR and CRAL models was analyzed by independent *t*-test values comparing the delta values of Lt.-GVS and Lt.-UL to the respective delta values of Rt.-GVS and Rt.-UL. *Significant difference between Lt.-UL and Lt.-GVS; #significant difference between Rt.-UL and Rt.-GVS; *, #*p* < 0.05; **, ##*p* < 0.01; ***, ###*p* < 0.001.

Locomotion reflected by the total path length on POD 3 (*p* < 0.001, Bonferroni test, for both Lt.- and Rt.- UL groups) and POD 7 (*p* < 0.001, Bonferroni test, for both Lt.- and Rt.- UL groups) (Figure 5A) and by spontaneous alternation (SAP) on POD 3 (Lt.-UL, *p* = 0.001; Rt.-UL, *p* < 0.001, Bonferroni test) and POD 7 (Lt.-UL, *p* = 0.007; Rt.-UL, *p* = 0.032, Bonferroni test) (Figure 5B) were improved markedly after GVS applications in both groups. The subgroup analysis between the Lt.- and Rt.-GVS groups revealed that GVS intervention was significantly more effective in the Rt.-UL mice than the Lt.-UL mice on POD 3 on total path length (*p* = 0.004, *F* = 0.54, independent *t*-test) (Figure 5A) and SAP (*p* = 0.02, *F* = 2.142, independent *t*-test) (Figure 5B).

Short-term spatial memory and attention as reflected by SAP, SAR, and PRT during the Y maze were also significantly improved after bipolar GVS intervention in both groups. The GVS protocol exhibited a positive effect on the recovery of SAP on POD 3 (Lt.-side, Δ = 6.17, *p* = 0.001; Rt.-side, Δ = 10.67, *p* < 0.001, Bonferroni test) and POD 7 (Lt.-side, Δ = 7.34, *p* = 0.007; Rt.-side, Δ = 6.68, *p* = 0.032, Bonferroni test). SAR showed a significant improvement after GVS intervention in both side groups on POD 3 (Lt.-side, *p* = 0.003; Rt.-side, *p* = 0.001, Mann–Whitney *U*-test) and in the left side group only on POD 7 (*p* = 0.003, Mann–Whitney *U*-test). The GVS intervention was significantly more effective in the Rt.-UL mice than the Lt.-UL mice on POD 7 (*p* < 0.001, *F* = 0, independent *t*-test) (Figure 5C). The PRT was significantly improved after GVS on POD 3 (Lt.-side, *p* < 0.001; Rt.-side, *p* = 0.005, Bonferroni test) and POD 7 (Lt.-side, *p* < 0.001, Bonferroni test). The subgroup analysis revealed that the GVS intervention was more effective in the Rt.-UL mice than the Lt.-UL mice on POD 3 (*p* = 0.029, *F* = 1.070, independent *t*-test) and POD 7 (*p* = 0.008, *F* = 3.706, independent *t*-test) (Figure 5D).

Long-term consolidative spatial memory reflected by the escape latency in MWM was improved after GVS in both groups on hidden platform TD 2 (*p* < 0.05, Bonferroni test, for both Lt.- and Rt.-sides), TD 3 (Lt.-side, *p* = 0.002; Rt.-side, *p* = 0.045, Bonferroni test), and TD 4 (Lt.-side, *p* < 0.001; Rt.-side, *p* = 0.002, Bonferroni test). There was, however, no noticeable difference between the Lt.-GVS and Rt.-GVS groups (Figure 5E). The probe trial on POD 14 showed substantial improvement after GVS intervention in both side groups (Lt.-side, *p* < 0.001; Rt.-side, *p* = 0.003, Bonferroni test), with a significant difference between the Lt.-GVS and Rt.-GVS groups (*p* = 0.034, *F* = 0.048, independent *t*-test) (Figure 5F).

## Discussion

Vestibular information has been demonstrated to have a major role in determining egocentric heading and rotations around an earth-vertical axis for accurate spatial performance (74–77). Given the critical function of the vestibular system in spatial orientation with three-dimensional coordinates (17, 30, 31, 78–80) *via* its multisensory, highly convergent, and highly multimodal mechanisms (10–13, 31), it is reasonable to speculate that unilateral vestibular lesions will affect spatial cognition, as shown in our previous study (22). Both right and left UVD impaired short-term spatial working memory and spatial reference memory during the week after UL, but the deficits recovered within 2 weeks. Long-term spatial cognition, as evidenced by the longer escape latency in hidden platform trials and a decrease in the percentage of time spent in the target quadrant in the probe trial at 2 weeks after UL, was impaired in the UVD groups compared with the control group. Although some processes are shared in the hippocampal CA1 subregion, the short-term and different phases of long-term memory are not sequentially linked, and the consolidation of new memory into long-term memory is time-dependent (66, 81–83). While short-term memory is formed almost immediately and is disrupted by subsequent learning, long-term memory requires consolidation over time as a result of hormonal and neurological influences on memory and the involvement of molecular and cellular mechanisms (66).

Our findings corroborated previous research indicating that acute UVD can cause spatial cognition deficiencies, especially transient short-term and more lasting long-term memory cognition deficits (18–20, 22, 84). Multiple pieces of evidence support our findings, including studies of electrical changes [electrical excitability (85–87), particularly long-term potentiation (86, 88)] and biochemical changes [neuronal nitric oxide synthase expression (23, 85, 89–91), N-methyl-D-aspartate receptor subunit expression (24), glucose metabolism (92), and glucocorticoid receptor expression (93)] and cellular proliferation [Arc, zif268, c-fos gene expression (51, 94, 95)] in the brain, in particular in the hippocampal formation, of UVD individuals both *in vitro* and *in vivo*. Several studies have revealed that these alterations can persist for an extended duration following UVD lesions, for 1 month (86) or 5–6 months (85), although the studies did not differentiate between short-term and long-term spatial memory deficits.

The vestibular system integrates multisensory signals, most notably vestibular and visual input (96), between the ipsilateral and contralateral sides of the multi-level brain regions. Despite the lack of animal data for comparison of behaviors between left- and right-sided UL animals, some results from human studies explain the current experimental findings as vestibular lateralization differences reflect evolutionary development between the two species (31, 41). The prevalence of hemispatial neglect and the frequency of spatial processing deficits were significantly higher in right-handers with right hemispheric strokes compared with those of left hemispheric strokes (97). In parallel, pusher syndrome, in which patients actively push away from the non-hemiparetic side and show postural imbalance (98), is observed with a significantly higher frequency in patients with right hemispheric stroke than in those with left hemispheric stroke, and slower recovery from pushing is shown after right hemispheric stroke (99). In addition, cortical and subcortical activation by vestibular caloric stimulation depends on the handedness of the individuals and the side of the stimulated ear, i.e., activation was bilateral but predominant in the hemisphere ipsilateral to the stimulated ear and exhibited right-hemispheric dominance in right-handers or left-hemispheric dominance in left-handers (31). With regard to the pathological state, a recent (18). F-fluorodeoxyglucose (F-FDG) PET study reported that brain activity in the acute phase of right- and left-sided UVD exhibits different compensatory patterns, in which the dominant ascending input is shifted from the ipsilateral to contralateral pathways, presumably due to the missing ipsilateral vestibular input (100). This might imply that the vestibular “dominant” right ear lesion might show more severe consequences than the vestibular “non-dominant” left ear lesion in humans (100, 101). Another study using H_2_O^15^-PET imaging demonstrated that the side-specific suppression of vestibular cortex activations was more pronounced in patients with right-sided thalamic lesions than those with left-sided lesions (37). These data demonstrated the functional importance of the dominance of ipsilateral vestibular ascending pathways from the end-organ (ear) to the relay-station (thalamus) and to the vestibular dominant right hemisphere in right-handed individuals (33, 37, 40).

Several human studies have investigated spatial cognition concerning the side of vestibular loss; however, their findings remain controversial (20, 44, 84, 100–102). One study demonstrated differential impairment of embodied spatial cognition between left- and right-sided UVD patients, with left-sided UVD patients more severely affected (44). However, other studies showed that the spatial memory and navigation of right-sided UVD patients were more severely affected (101, 103). The right-sided UVD patients performed significantly worse in the probe trial of virtual MWM, i.e., spending less time and distance searching in the correct quadrant and having a higher heading error than the left-sided UVD patients or controls (101). These findings are compatible with the recently described dominance of the right labyrinth and the vestibular cortex in the right hemisphere (37, 100, 101). This discrepancy can be explained by the variety of spatial cognitive tasks in the study that were driven by distinct brain areas that are lateralized at various levels (44).

Vestibular lateralization studies in animal models such as rodents have been promoted. A recent microPET study revealed that the vestibular processing in rats follows a strong left hemispheric dominance independent from the “handedness” of the animals (41). The authors showed that the left vestibular information was processed by a complex cortical and subcortical network, whereas the right vestibular input was processed by fewer cortical areas, which suggested dominant left-sided vestibular information processing (41). Phylogenetic concepts have led to the hypothesis that the difference in vestibular dominance between rodents and humans is the result of the evolution of speech and handedness in humans, both of which represent mechanisms that could have led to reconfiguration within the vestibular cortical network (31, 41). Considering the vestibular lateralization of the left hemisphere in rodents (41), we hypothesize that the missing dominant ipsilateral input resulted in a more serious outcome than those occurring in missing input of the non-dominant side.

In our previous study, we allocated the cathode to the lesion side (right) and the anode to the intact side (left) for GVS, which resulted in a significant improvement in spatial cognition as well as locomotion and VOR in the UL mouse model (22, 49). In the current study, we used the same protocol for GVS (CLAR in the Lt.-UL and CRAL in the Rt.-UL groups), which helped to rebalance the firing rate with attenuating the intact side and facilitating the lesioned side (26, 28) and accelerate vestibular recovery. Although this study corroborated previous findings of the effects of GVS in improving UVD-induced spatial cognition impairments (22, 104), the precise mechanism remains unknown. However, increasing evidence has shown that GVS enhances the function of spatial navigation through multimodal mechanisms (22). The GVS currently likely operates by modulating the firing rate of vestibular afferents (26, 28), and GVS also excites medial vestibular nuclei and increases the firing rates of hippocampal CA1 complex spike cells corresponding to place cells (105). The electrical stimulation of afferent vestibular fibers evidently enhances the long-term potentiation and long-term depression in the vestibular nuclei of rats *in vitro* (106–109), which in turn facilitates the effects in the hippocampus. Similarly, GVS has been shown to generate theta activity in numerous areas of the hippocampal formation (110), which plays a pivotal role in spatial information processing and modulates self-movement signals (111). This formation also improves neuronal activity for spatial orientation (112, 113). Additionally, an increase of c-Fos-positive cells in the hippocampus, which is an indicator of neuronal activation, was detected following subsequent repetition of GVS (29, 95).

Our findings showed, for the first time, the differential effects of GVS intervention depending on the lesion sites. The Lt.-UL mice group showed greater improvements after GVS intervention in both short- and long-term spatial memory, which might be due to poor performance in the Lt.-UL mice compared with the Rt.-UL mice before GVS application. Alternatively, this selective effect was due to the results of a functional asymmetry of the vestibular apparatus and a set of common neural mechanisms between each GVS paradigm (CRAL vs. CLAR) and brain regions (114). In a study with healthy subjects with functional MRI (fMRI), the CLAR activated both hemispheres, whereas the CRAL activated only the right hemisphere (115). However, there might be differences between the GVS protocols as well as between patients with UVD and normal individuals; (116) this should be examined in future studies.

In conclusion, our results showed that right- and left-sided UVD impairs spatial cognition and locomotion differently and exhibits different compensatory patterns in mice. Considering that the vestibular processing in rodents follows a strong left hemispheric dominance, ipsilateral peripheral vestibular injuries on the dominant side have more severe consequences and slower recoveries than those on the non-dominant side. We also identified the effects of bipolar GVS on accelerating recovery for UVD-induced spatial cognition when the cathode (stimulating) was assigned to the lesion side. The current findings suggest that sequential bipolar GVS intervention might have substantial implications for comprehensive and specialized management in patients with impairment of spatial cognition, especially induced by unilateral peripheral vestibular damage on the dominant side.

## Data Availability Statement

The original contributions presented in the study are included in the article/supplementary material, further inquiries can be directed to the corresponding author/s.

## Ethics Statement

The animal study was reviewed and approved by the Animal Care Committee of Gachon University of Medicine and Science (IRB MRI2019-0008).

## Author Contributions

S-YO involved in study concept and design and drafting a significant portion of the manuscript. TTN, G-SN, and J-JK involved in acquisition and analysis of data and drafting a significant portion of the manuscript and figures. GCH and J-SK involved in acquisition and analysis of data. J-SK and MD involved in analysis of data and drafting a significant portion of the manuscript. All authors contributed to the article and approved the submitted version.

## Funding

This work was supported by a National Research Foundation of Korea (NRF) grant funded by the Korean government (Ministry of Science and ICT) (No. 2019R1A2C1004796), and by the BK21 FOUR Program by Jeonbuk National University Research Grant.

## Conflict of Interest

The authors declare that the research was conducted in the absence of any commercial or financial relationships that could be construed as a potential conflict of interest.

## Publisher's Note

All claims expressed in this article are solely those of the authors and do not necessarily represent those of their affiliated organizations, or those of the publisher, the editors and the reviewers. Any product that may be evaluated in this article, or claim that may be made by its manufacturer, is not guaranteed or endorsed by the publisher.
